# Coronavirus disease 2019 infection among working-aged people with multiple sclerosis and the impact of disease-modifying therapies

**DOI:** 10.1177/20552173241248293

**Published:** 2024-04-27

**Authors:** Chantelle Murley, Emma Pettersson, Jan Hillert, Alejandra Machado, Emilie Friberg

**Affiliations:** Division of Insurance Medicine, Department of Clinical Neuroscience, 27106 Karolinska Institutet, Stockholm, Sweden; Division of Neurology, Department of Clinical Neuroscience, 27106 Karolinska Institutet, Stockholm, Sweden; Division of Insurance Medicine, Department of Clinical Neuroscience, 27106 Karolinska Institutet, Stockholm, Sweden

**Keywords:** Coronavirus disease 2019, multiple sclerosis, disease-modifying therapy, long coronavirus disease 2019, pandemic, drug therapy, epidemiology, Sweden

## Abstract

**Background:**

The risk of coronavirus disease 2019 among people with multiple sclerosis with different disease-modifying therapies is not well established.

**Objective:**

To investigate the occurrence of coronavirus disease 2019 and the remaining symptoms among people with multiple sclerosis and the associations with different disease-modifying therapies.

**Methods:**

Individuals aged 20–50 listed in the Swedish Multiple Sclerosis Registry were invited to participate in a survey in 2021. Information on reported coronavirus disease 2019 infection and remaining symptoms were linked to individual-level register data. The risks by disease-modifying therapy of having coronavirus disease 2019 or having remaining symptoms were estimated with logistic regression.

**Results:**

Of the 4393 participants, 1030 (23.4%) self-reported coronavirus disease 2019 (749 confirmed and 281 suspected). The observed odds for coronavirus disease 2019 did not differ by disease-modifying therapy (*p*-values <0.05). The majority reporting coronavirus disease 2019 had fully recovered (68.5%), 4.2% were currently/recently sick, and 27.0% had symptoms remaining after 2 months. The most frequently reported remaining symptoms involved one's sense of smell or taste (37.0%), fatigue (20.0%), and breathing (12.0%). No statistically significant associations were observed between having remaining symptoms and the disease-modifying therapy.

**Conclusion:**

Despite the initial concerns of differing infection risks by MS treatments, we observed no differences in coronavirus disease 2019 occurrence or remaining symptoms among those who had coronavirus disease 2019. Nonetheless, exercising caution in interpreting our findings, it remains implicit that people with multiple sclerosis are particularly susceptible to infection and that lingering symptoms may persist beyond the initial infection.

## Introduction

Many people with multiple sclerosis (PwMS) faced uncertainty regarding their risk of coronavirus disease 2019 (COVID-19).^1^ These concerns are related both to reducing their risk of infection with COVID-19 and also regarding receiving treatment or care for multiple sclerosis (MS).^2^ PwMS generally have an elevated risk for serious infections, especially those with progressive forms of the disease.^3^ Furthermore, general risks of infection are also partly dependent on MS treatment choice,^4^ owing to the different mechanisms to suppress the immune response that disease-modifying therapies (DMTs) for MS invoke.^5^ Differences in dosing frequencies and locations for administration of DMTs can also affect contagion risk. Hence, raising questions over whether the risk of infection of COVID-19 differed by DMT.

COVID-19 symptoms vary from asymptomatic to severe,^6^ with severe morbidity often attributed to an exaggerated immune response rather than unchecked viral replication.^7^ Long-COVID includes the presence of new or lingering symptoms beyond initial infection.^8^ While long-COVID only affects a proportion of individuals, it imposes an increasing burden on individuals and society.^8–10^

Our knowledge of COVID-19 and its impact on PwMS has evolved over time.^7,11^ While early insights suggested slight differences in the risk of infection and disease severity related to COVID for PwMS compared to the general population,^7^ recent studies suggest similar rates of COVID-19 infection.^12^ Factors such as older age, obesity, comorbidities, progressive MS, severe physical disability, and recent steroid use contribute to worse outcomes.^13–16^ Findings regarding the use of DMTs are mixed but suggest fewer differences in risk of infection or severity than initially hypothesised based on mode of action and pre-existing knowledge on general infection risks by DMT.^7,11,17^ Recent studies suggest that only anti-CD20 monoclonal antibodies may moderately increase COVID-19 severity.^1,14,15^ Treatment considerations are crucial for MS management and treatment protocols in Sweden, where there is a high off-label use of rituximab, an anti-CD20 monoclonal antibody.^18^

In Sweden, where MS prevalence is high,^19^ a unique strategy was adopted during the pandemic, primarily relying on public health recommendations and voluntary behaviour change.^20^ Despite studies already being published, variations in COVID-19 incidence rates across countries exist owing to implemented non-pharmaceutical interventions and MS clinical management approaches.^21^ Accordingly, there is an interest in exploring the impact of COVID-19 on PwMS in different contexts.

Hence, we aimed to investigate the proportion of working-aged PwMS in Sweden having had confirmed or suspected COVID-19 as well as any remaining symptoms and whether the type of MS treatment was associated with the occurrence of COVID-19 or remaining symptoms.

## Materials and methods

A cross-sectional study was conducted using questions related to COVID-19 infection and remaining symptoms from a survey as well as individual-level linked register data.

### Data sources and study population

All individuals listed in the Swedish MS Registry (SMSreg)^22^ that were 20–50 years old and living in Sweden, were invited to participate between May and September 2021 in an online survey administered by Statistics Sweden. Of the 8458 PwMS invited, 4412 (52%) answered the survey. Further details of the study population are published elsewhere.^23^ Statistics Sweden linked the survey responses to individual-level clinical data from the SMSreg and demographic data from the Longitudinal Integrated Database for Health Insurance and Labor Market Studies (LISA).^24^ The anonymised data was then delivered to the researchers.

### Variables

Three survey questions were used as outcomes in this study.

First, the proportion of all study participants who had COVID-19 was investigated with the question “*Do you or have you had COVID-19*?” with four possible responses:

- *Yes, and it is confirmed with a test*
- *Yes, I think so but have not had it confirmed with a test*
- *No*
- *I do not know*

Confirmed or suspected responses were also combined as a single alternative to represent having had COVID-19. This was based on the changing Swedish recommendations throughout the pandemic on who should be offered testing, as the default was not always mandatory mass testing and reporting if symptomatic*.*^25^

Second, among individuals responding that they have had confirmed or suspected COVID-19, there was a follow-up question about their recovery ‘How do you feel now?’ with three possible responses:

- *I am sick/less than two months since I fell ill*
- *Fully recovered*
- *Remaining symptoms*.

Third, among the participants responding that they had remaining symptoms, an open-ended response was available to the question ‘What remaining symptoms do you have*?’.* The remaining symptoms in their statements were then grouped into the following symptom clusters that emerged from the data:

(a) Breathing
(b) Physical
(c) Cognition
(d) Sensory/feel
(e) Smell and taste
(f) Impaired immune system/susceptibility to infection
(g) Sensory/vision
(h) Pain and aches
(i) Fatigue/lack of energy
(j) Other
(k) Missing

The exposure of interest was the participants’ DMT. This was informed by the most recent DMT from the SMSreg*.* Glatiramer acetate and interferons were grouped as injectables.^26^ Accordingly, the DMT categories were: *No treatment, Alemtuzumab, Cladribine, Dimethyl fumarate, Fingolimod, Injectables, Haematopoietic stem cell transplantation (HSCT), Natalizumab, Ocrelizumab, Rituximab, and Teriflunomide.*

Lastly, the following covariates were included from register data: Age groups (20–29; 30–39; 40–49; and 50–51), legal sex (women, men), country of birth (Sweden: yes, no), level of education (primary school, high school, university), type of living area (city, town/suburb, rural area) and type of MS (relapsing-remitting MS, primary progressive MS, secondary progressive MS, missing).

### Statistical analyses

The study population was described by frequencies and proportions for all, and by their reported COVID-19 status. Chi-square tests were then used to test for differences in proportions reporting COVID-19 comparing those with and without COVID-19, and in contrast to those responding do not know.

The risk of having COVID-19 by DMT was estimated using logistic regression in unadjusted and mutually-adjusted models including all covariates except type of MS and then lastly, all covariates including type of MS. Models were run with dimethyl fumarate as the reference DMT. An alternative model specified rituximab as the reference given its wide use in Sweden^18^ and the particular interest owing to potentially heightened vulnerability to infection with this DMT.^27^ The results are reported as odds ratios (ORs) with 95% confidence intervals (CIs). The main analysis included those reporting Yes, both confirmed with a test and suspected, with sensitivity analyses investigating only those reporting COVID-19 confirmed by a test.

Among the participants having reported that they have had COVID-19, the proportion having remaining symptoms and their symptom clusters were then explored with frequencies and proportions.

Lastly, the risks of having remaining symptoms (yes, no) with a given DMT were explored with logistic regression and reported as ORs with 95% CIs with unadjusted and adjusted models including all covariates.

All statistical analyses were performed using R version 4.2.2 and a p-value of <0.05 was considered statistically significant.

## Results

Participants who answered the question regarding having had COVID-19 (*n* = 4409) and had valid information on their most recent treatment (which DMT or no treatment) in the SMSreg (*n* = 4393) were included in the study. Of these 4393 working-aged participants with MS, 71.3% were women, 88.1% were born in Sweden, and 60.4% had University-level education (Table 1). Nearly all (91.5%) had relapsing-remitting MS. The most common current DMTs were rituximab (48.4%), natalizumab (12.4%), and dimethyl fumarate (10.5%). No treatment (7.6%), injectables (6.6%, of which 66.7% interferons and 33.3% glatiramer acetate) and fingolimod (5.9%) and the remaining treatment options were less frequent.

**Table 1.** Study population characteristics for all participants and by reported COVID-19 status.

	All participants	Do you or have you had COVID-19?						
		Yes (confirmed/suspected)	Yes, and it is confirmed with a test	Yes, I think so but have not had it confirmed with a test	No	I do not know	Yes vs No	Yes vs No vs I do not know
	(*N* = 4393)	(*N* = 1030)	(*N* = 749)	(*N* = 281)	(*N* = 2881)	(*N* = 482)	*p*-value	*p*-value
*Age*							0.266	**0**.**045**
20–29	390 (8.9%)	106 (10.3%)	74 (9.9%)	32 (11.4%)	240 (8.3%)	44 (9.1%)		
30–39	1402 (31.9%)	312 (30.3%)	224 (29.9%)	88 (31.3%)	919 (31.9%)	171 (35.5%)		
40–49	2220 (50.5%)	520 (50.5%)	381 (50.9%)	139 (49.5%)	1459 (50.6%)	241 (50.0%)		
50–51^a^	381 (8.7%)	92 (8.9%)	70 (9.3%)	22 (7.8%)	263 (9.1%)	26 (5.4%)		
*Sex*							0.575	**<0**.**001**
Woman	3134 (71.3%)	740 (71.8%)	542 (72.4%)	198 (70.5%)	2096 (72.8%)	298 (61.8%)		
Man	1259 (28.7%)	290 (28.2%)	207 (27.6%)	83 (29.5%)	785 (27.2%)	184 (38.2%)		
*Country of birth*							0.056	**0**.**011**
Sweden	3871 (88.1%)	895 (86.9%)	655 (87.5%)	240 (85.4%)	2567 (89.1%)	409 (84.9%)		
Other^b^	522 (11.9%)	135 (13.1%)	94 (12.6%)	41 (14.6%)	314 (10.9%)	73 (15.1%)		
*Level of education*							**0**.**011**	**<0**.**001**
Primary school^c^	207 (4.7%)	63 (6.1%)	40 (5.3%)	23 (8.2%)	113 (3.9%)	31 (6.4%)		
High school	1531 (34.9%)	357 (34.7%)	265 (35.4%)	92 (32.7%)	984 (34.2%)	190 (39.4%)		
University	2655 (60.4%)	610 (59.2%)	444 (59.3%)	166 (59.1%)	1784 (61.9%)	261 (54.1%)		
*Type of living area*							**0**.**010**	**0**.**003**
City	1956 (44.5%)	500 (48.5%)	353 (47.1%)	147 (52.3%)	1249 (43.4%)	207 (42.9%)		
Town/suburb	1694 (38.6%)	373 (36.2%)	278 (37.1%)	95 (33.8%)	1112 (38.6%)	209 (43.4%)		
Rural area^d^	743 (16.9%)	157 (15.2%)	118 (15.8%)	39 (13.9%)	520 (18.0%)	66 (13.7%)		
*Type of MS*							0.105	0.214
Relapsing remitting MS	4021 (91.5%)	963 (93.5%)	698 (93.2%)	265 (94.3%)	2622 (91.0%)	436 (90.5%)		
Primary progressive MS	81 (1.8%)	14 (1.4%)	10 (1.3%)	4 (1.4%)	54 (1.9%)	13 (2.7%)		
Secondary progressive-MS	242 (5.5%)	44 (4.3%)	34 (4.5%)	10 (3.6%)	171 (5.9%)	27 (5.6%)		
Missing	49 (1.1%)	9 (0.9%)	7 (0.9%)	2 (0.7%)	34 (1.2%)	6 (1.2%)		
*Latest DMT*							0.850	0.471
No treatment	336 (7.6%)	72 (7.0%)	50 (6.7%)	22 (7.8%)	216 (7.5%)	48 (10.0%)		
Alemtuzumab	49 (1.1%)	12 (1.2%)	7 (0.9%)	5 (1.8%)	34 (1.2%)	3 (0.6%)		
Cladribine	76 (1.7%)	14 (1.4%)	12 (1.6%)	2 (0.7%)	54 (1.9%)	8 (1.7%)		
Dimethyl fumarate	461 (10.5%)	118 (11.5%)	92 (12.3%)	26 (9.3%)	299 (10.4%)	44 (9.1%)		
Fingolimod	258 (5.9%)	61 (5.9%)	41 (5.5%)	20 (7.1%)	168 (5.8%)	29 (6.0%)		
Injectables	288 (6.6%)	63 (6.1%)	50 (6.7%)	13 (4.6%)	190 (6.6%)	35 (7.3%)		
HSCT	87 (2.0%)	14 (1.4%)	10 (1.3%)	4 (1.4%)	62 (2.2%)	11 (2.3%)		
Natalizumab	546 (12.4%)	129 (12.5%)	98 (13.1%)	31 (11.0%)	359 (12.5%)	58 (12.0%)		
Ocrelizumab	48 (1.1%)	10 (1.0%)	8 (1.1%)	2 (0.7%)	27 (0.9%)	11 (2.3%)		
Rituximab	2128 (48.4%)	512 (49.7%)	368 (49.1%)	144 (51.2%)	1393 (48.4%)	223 (46.3%)		
Teriflunomide	116 (2.6%)	25 (2.4%)	13 (1.7%)	12 (4.3%)	79 (2.7%)	12 (2.5%)		
*How do you feel now?*								
I am sick*/*less than two months since I fell ill	43 (1.0%)	43 (4.2%)	31 (4.1%)	12 (4.3%)	0 (0%)	0 (0%)		
Fully recovered	706 (16.1%)	706 (68.5%)	488 (65.2%)	218 (77.6%)	0 (0%)	0 (0%)		
Remaining symptoms	278 (6.3%)	278 (27.0%)	227 (30.3%)	51 (18.1%)	0 (0%)	0 (0%)		
Missing	3366 (76.6%)	3 (0.3%)	3 (0.4%)	0 (0%)	2881 (100%)	482 (100%)		

*P*-values from Chi^2^ tests. Statistically significant *p*-values are highlighted in bold font. In the ‘yes’ category, participants comprise those with confirmed or suspected cases of COVID-19. In contrast, the ‘no’ category encompasses individuals who are either certain not to have had COVID-19 or are uncertain about their COVID-19 status. DMT: disease-modifying therapy; HSCT: haematopoietic stem cell transplantation; MS: multiple sclerosis; COVID-19: coronavirus disease 2019.

^a^ Participants could have turned 51 by the time they completed the survey.

^b^ Individuals with missing information were assigned to other.

c Individuals with missing information were assigned to primary school.

^d^ Individuals with missing information were assigned to rural area.

In total, 1030 (23.4%) of the participants responded that they had COVID-19, whether confirmed (*n* = 749) or suspected (*n* = 281), 2881 (65.6%) reported not having had COVID-19, and 482 (11.0%) did not know. Differences in the proportions reporting that they have had COVID-19 (both confirmed or suspected) compared with those responding “No”), were observed by educational level and the type of living area.

The risk of COVID-19 was investigated among the 3911 participants who responded that they have had COVID-19 (confirmed or suspected) compared to those who had not had it. The ORs for having had COVID-19 were similar across the different DMTs (Table 2). Using dimethyl fumarate as the reference treatment, the observed ORs were all less than 1, although there were no statistically significant differences (Table 2). ORs closer to 1 were observed after adjusting for potential confounders. Similarly, using rituximab (the most common DMT) as the reference group, no differences in the risk of COVID-19 were observed by type of treatment (Supplemental Material, Table 1). The findings did not substantially change when investigating the risk only among the participants with confirmed COVID-19 compared to those not having had it (*n* = 3630) (Supplemental Material, Table 2).

**Table 2.** Odds ratios for having had COVID-19 (both confirmed with a test or suspected) compared with not having had COVID-19 in participants with MS (*n* = 3911).

Characteristic	Unadjusted		Adjusted^a^		Adjusted^b^	
	OR (95% CI)^c^	*p*-value	OR (95% CI)^c^	*p*-value	OR (95% CI)^c^	*p*-value
*Latest DMT*						
Dimethyl fumarate	—		—		—	
No treatment	0.84 (0.60–1.19)	0.332	0.86 (0.60–1.21)	0.379	0.93 (0.65–1.32)	0.689
Alemtuzumab	0.89 (0.43–1.74)	0.752	0.87 (0.42–1.71)	0.706	0.87 (0.42–1.70)	0.700
Cladribine	0.66 (0.34–1.20)	0.188	0.64 (0.33–1.17)	0.164	0.65 (0.33–1.18)	0.172
Fingolimod	0.92 (0.64–1.32)	0.652	0.92 (0.63–1.32)	0.637	0.92 (0.64–1.32)	0.660
Injectables	0.84 (0.59–1.20)	0.337	0.86 (0.60–1.22)	0.397	0.86 (0.60–1.23)	0.425
HSCT	0.57 (0.30–1.03)	0.077	0.58 (0.30–1.04)	0.082	0.61 (0.32–1.11)	0.121
Natalizumab	0.91 (0.68–1.22)	0.531	0.91 (0.68–1.22)	0.528	0.92 (0.68–1.23)	0.563
Ocrelizumab	0.94 (0.42–1.94)	0.869	0.94 (0.42–1.95)	0.875	0.94 (0.42–1.95)	0.876
Rituximab	0.93 (0.74–1.18)	0.555	0.93 (0.73–1.18)	0.528	0.95 (0.75–1.21)	0.668
Teriflunomide	0.80 (0.48–1.30)	0.385	0.81 (0.48–1.31)	0.397	0.81 (0.48–1.31)	0.398
*Age (years)*						
20–29	—		—		—	
30–39	**0.77** (**0.59–1.00)**	**0**.**049**	0.79 (0.61–1.04)	0.086	0.79 (0.61–1.04)	0.091
40–49	0.81 (0.63–1.04)	0.092	0.86 (0.67–1.11)	0.249	0.88 (0.69–1.15)	0.352
50–51	0.79 (0.57–1.10)	0.166	0.84 (0.60–1.17)	0.307	0.88 (0.63–1.24)	0.476
*Sex*						
Woman	—		—		—	
Man	1.05 (0.89–1.23)	0.575	1.02 (0.86–1.19)	0.841	1.02 (0.87–1.20)	0.789
*Country of birth*						
Sweden	—		—		—	
Other	1.23 (0.99–1.53)	0.057	1.16 (0.93–1.45)	0.183	1.17 (0.94–1.46)	0.159
*Level of education*						
Primary school	—		—		—	
High school	**0.65** (**0.47–0.91)**	**0**.**011**	**0.68** (**0.49–0.96)**	**0**.**025**	**0.67** (**0.48–0.95)**	**0**.**021**
University	**0.61** (**0.45–0.85)**	**0**.**003**	**0.62** (**0.45–0.87)**	**0**.**004**	**0.61** (**0.44–0.85)**	**0**.**003**
*Type of living area*						
City	—		—		—	
Town/suburb	**0.84** (**0.72–0.98)**	**0**.**027**	**0.84** (**0.71–0.99)**	**0**.**032**	**0.84** (**0.72–0.99)**	**0**.**035**
Rural area	**0.75** (**0.61–0.93)**	**0**.**007**	**0.74** (**0.60–0.92)**	**0**.**006**	**0.75** (**0.60–0.92)**	**0**.**008**
*Type of MS*						
Relapsing remitting MS	—				—	
Primary progressive MS	0.71 (0.38–1.24)	0.249			0.72 (0.38–1.27)	0.282
Secondary progressive MS	**0.70** (**0.49–0.97)**	**0**.**040**			**0.68** (**0.47–0.96)**	**0**.**032**
Missing	0.72 (0.32–1.44)	0.385			0.73 (0.33–1.47)	0.403

CI: confidence interval; DMT: disease-modifying therapy; HSCT: haematopoietic stem cell transplantation; MS: multiple sclerosis; OR: odds ratio; COVID-19: coronavirus disease 2019.

^a^ Mutually-adjusted for all other covariates except type of MS.

^b^ Mutually-adjusted for all other covariates including type of MS.

^c^ OR with 95% CIs from logistic regression models.

Nevertheless, differences in the occurrence of COVID-19 were observed for characteristics other than DMT choice. Smaller ORs for COVID-19 by increasing levels of education and less populated living areas (*p*-values <0.05) among the participants with MS were observed in the unadjusted and adjusted analyses (Table 2). In addition, participants with secondary-progressive MS had a 32% decreased occurrence of COVID-19 compared with participants with relapsing-remitting MS (OR: 0.68; 95% CI: 0.47–0.96).

Among 1030 who reported that they have had COVID-19 (both confirmed or suspected), the majority had fully recovered (68.5%), 4.2% were sick or it had been less than 2 months when they responded, and 27.0% reported that they had remaining symptoms (Table 1). The contents of the participants’ statements of their remaining symptoms were categorised into 11 symptom clusters (Table 3). The three most frequent remaining symptoms among related to the sense of smell or taste (37%), fatigue/lack of energy (20.0%), and breathing (12.0%). No differences in the risk of having any remaining symptoms (*n* = 278) compared to being fully recovered (*n* = 706) were observed by DMT when dimethyl fumarate was the comparator (Table 4).

**Table 3.** Clusters of symptoms identified from the statements of participants who reported having had coronavirus disease 2019 (COVID-19) and having remaining symptoms at least 2 months later, for all (*n* = 278).

Symptom cluster	Overall n (%)
**Breathing** (difficulty breathing, shortness of breath, pain in the airways, increased difficulty breathing when exerting oneself, pressure in the chest, heavy breathing, worse wheezing, cough, mucus in the throat or lungs, decreased lung function, sensitive lungs, etc.)	32 (12)
**Physical** (walking ability, motor disorders, problems with physical exertion/activity, reduced function and stamina, dizziness, increased pulse/heart palpitations (dysautonomia, high pulse))	23 (8.3)
**Cognition** (fatigue, brain fatigue)	16 (5.8)
**Sensation** (numbness, burning sensation, sensory impact, hemiparesis or any degree of weakness)	10 (3.6)
**Smell and taste** (impaired, loss or absence of taste or sense of smell, etc.)	102 (37)
**Pain and aches** (headache, chest pain, sore throat, muscle pain, joint pain)	17 (6.1)
**Fatigue**/**lack of energy** (weak in the body, etc)	56 (20)
Other* (fever, stomach, bowel and bladder problems, stroke, impaired immune system/susceptibility to infection, sensory/vision (inflammation of the optic nerve visual impairment, eye problems in general), unspecified complaints, do not want to state one's complaints, missing)	22 (7.9)

*Impaired immune system/susceptibility to infection (*n* = 4), sensory/vision (*n* = 7) and missing (*n* = 3) are integrated into ‘Other’ category.

**Table 4.** Risk of remaining symptoms after COVID-19 (confirmed with a test or suspected) (*n* = 984).

Characteristic	Unadjusted		Adjusted^a^	
	OR (95% CI)^b^	*p*-value	OR (95% CI)^b^	*p*-value
*Latest DMT*				
Dimethyl fumarate	—		—	
No treatment	0.99 (0.49–1.94)	0.967	0.85 (0.41–1.72)	0.657
Alemtuzumab	0.56 (0.08–2.28)	0.469	0.56 (0.08–2.35)	0.482
Cladribine	1.75 (0.49–5.67)	0.361	1.67 (0.47–5.50)	0.405
Fingolimod	0.76 (0.34–1.61)	0.486	0.77 (0.34–1.64)	0.504
Injectables	1.26 (0.63–2.51)	0.505	1.27 (0.63–2.54)	0.500
HSCT	0.76 (0.16–2.65)	0.692	0.74 (0.16–2.66)	0.674
Natalizumab	1.03 (0.58–1.85)	0.914	1.03 (0.57–1.85)	0.930
Ocrelizumab	1.20 (0.25–4.62)	0.804	1.06 (0.21–4.16)	0.941
Rituximab	1.20 (0.76–1.94)	0.442	1.18 (0.74–1.91)	0.495
Teriflunomide	0.93 (0.31–2.47)	0.890	0.90 (0.30–2.42)	0.834
*Age (years)*				
20–29	—		—	
30–39	0.98 (0.59–1.64)	0.924	1.03 (0.62–1.76)	0.900
40–49	1.07 (0.67–1.74)	0.792	1.12 (0.68–1.86)	0.664
50–51	1.17 (0.62–2.20)	0.625	1.24 (0.64–2.41)	0.514
*Sex*				
Woman	—		—	
Man	0.85 (0.62–1.16)	0.317	0.84 (0.60–1.15)	0.279
*Country of birth*				
Sweden	—		—	
Other	1.21 (0.81–1.80)	0.340	1.27 (0.83–1.92)	0.268
*Level of education*				
Primary school	—		—	
High school	0.68 (0.38–1.24)	0.200	0.64 (0.35–1.18)	0.144
University	0.61 (0.35–1.10)	0.094	0.58 (0.32–1.06)	0.072
*Type of living area*				
City	—		—	
Town/suburb	1.35 (1.00–1.83)	0.052	1.36 (1.00–1.87)	0.051
Rural area	1.09 (0.72–1.64)	0.674	1.07 (0.69–1.64)	0.748
*Type of MS*				
Relapsing remitting MS	—		—	
Primary progressive MS	1.05 (0.29–3.17)	0.933	1.05 (0.28–3.25)	0.937
Secondary progressive MS	**2.00** (**1.07–3.68)**	**0**.**027**	1.87 (0.97–3.57)	0.057
Missing	0.88 (0.13–3.83)	0.872	0.72 (0.10–3.22)	0.689

CI: confidence interval; DMT: disease-modifying therapy; HSCT: haematopoietic stem cell transplantation; MS: multiple sclerosis; OR: odds ratio; COVID-19: coronavirus disease 2019.

^a^ Mutually-adjusted for all other covariates including type of MS.

^b^ OR with 95% CIs from logistic regression models.

## Discussion

In this cross-sectional study of working-aged PwMS in Sweden, almost a quarter (23.4%) self-reported having had COVID-19 by summer 2021. No differences were observed among PwMS reporting COVID-19 by DMT, although higher education levels, living in less populous areas, and secondary-progressive MS were associated with a lower occurrence of COVID-19. A third (27.0%) of the participants with MS reporting COVID-19 also reported having symptoms remaining at least 2 months after their acute infection. Importantly, no differences were observed by DMT in the odds of reporting remaining symptoms. Eleven clusters of remaining symptoms were identified, the three most frequently reported related to the sense of smell or taste (37%), fatigue/lack of energy (20.0%), and breathing (12.0%). This real-world observational study on outcomes of COVID-19 in young and actively treated PwMS in Sweden adds to the body of knowledge on the impact of COVID-19 in the MS population.

The proportion of self-reported COVID-19 cases among PwMS participating in the study, even if only considering those with a confirmed positive test (17.1%), is higher than what was estimated for the whole Swedish population (11%) – based on the available data of the accumulated confirmed cases of all age groups and Swedish population on the 30 September, 2021.^28,29^ These findings could suggest a potential susceptibility for COVID-19 infection among PwMS, at least among those with the characteristics of the participants in this study (i.e., working-age population, living in highly and moderately populated-density areas, having relapsing-remitting MS, and actively treated – half of whom had rituximab). However, it's important to approach these findings with caution, as age groups are not directly comparable and younger populations in general, are recognised to face elevated risks against COVID-19.^30^ Additionally, and contrary to our findings, a recent systematic review and meta-analysis suggested a pooled infection rate of COVID-19 of 4% among PwMS with mean age ranged from 35 to 54 years.^31^ Despite this, the authors stated that incidence among PwMS differed significantly among countries, and Sweden, with its unique approach, was not included in this review.

Nevertheless, we found no statistically significant indications of a difference in risk of having COVID-19 by DMT, irrespective of the comparator. Our findings are consistent with other studies where the DMT was not a predictor for a severe COVID-19 course.^32^ Further, although not statistically significant, COVID-19 infection (confirmed by a test or clinically suspected) was most frequently observed among those on rituximab.^33^ Regardless, it seems that the choice of treatment among PwMS does not have a significant impact on the risk of contracting COVID-19.^7^

Besides, the participants may have also adjusted their life situation to correspond to their perceived elevated risk by adopting safer behaviour, e.g., physical distancing during the pandemic. This could partly explain our finding that participants with secondary-progressive MS had a decreased occurrence of COVID-19 compared with participants with relapsing-remitting. However, we did not capture such lifestyle changes in activities or health behaviours in the survey. Nonetheless, it can be hypothesised that individuals could not entirely control or mitigate their exposure owing to other factors, for example, having to go to their physical workplace or living with children, irrespective of self-identifying as belonging to a risk group.^34^ Further studies regarding family life and work-related situations among PwMS during the pandemic could aid in unravelling these questions.

An important contribution of our study is the insights on the proportion of PwMS having remaining symptoms after the acute infection. Similar to other studies, we identified the most frequent clusters of symptoms experienced included respiratory manifestations, loss of sense of smell or taste, and fatigue or brain fog.^8,9^ Our findings indicate that just under a third of the PwMS who had COVID-19 self-reported remaining symptoms after 2 months*.* Similar findings have been suggested in recent European studies, with 24%–30% of PwMS experiencing remaining COVID-19 symptoms for 1–3 months post-infection and decreasing rates thereafter.^17,35,36^ Estimates of long-COVID are varied,^9^ with many different definitions and lengths of time since initial symptoms are used in estimates across studies.^8,10^ We show that among PwMS, alongside previous studies in the general population, post-infection symptoms can occur among young individuals. Importantly, our findings suggest that the choice of DMT does not predispose an individual to having remaining symptoms after the initial COVID-19 infection.^17^

There are several methodological aspects to consider with our findings. First, we included both individuals with COVID-19 confirmed by a test result and suspected, like in other studies.^14^ Furthermore, we found no differences when limiting the risk of infection to only those with test results confirming COVID-19, as in a recent study.^33^ Our outcome of COVID-19 infection likely includes a higher proportion of less severe cases than in many other studies using clinically identified COVID-19. By including self-reported suspected COVID-19, our study inclusion was not dependent upon requiring healthcare and introducing ascertainment bias.^16^ In addition, no fatal cases were possible in our study as all were alive post-COVID-19 to respond to the survey. Nevertheless, this study comprises a representative selection of the population with MS in Sweden, considering the high coverage of MS patients in the SMSreg,^22^ and compared to other studies that are primarily focused on clinical settings with more active MS cases. While we lacked information regarding the vaccination status of the participants, the survey was conducted in the early stages of the vaccine roll-out. In addition, the strains of the virus circulating, and immune response are also relevant to consider. Finally, residual confounding is possible. We did not have information on all clinical and behavioural factors that could have affected the risk of COVID-19 infection at the time including obesity, hypertension, smoking, and other treatments such as recent steroid use.

To conclude, in this cross-sectional survey with linked register data, we observed no major differences in the risks of infection with COVID-19 by MS treatment, nor did we observe differences by MS treatment among participants reporting remaining symptoms following their acute infection. In total, a quarter of the participants with MS living in Sweden reported having had suspected or confirmed COVID-19 by summer 2021, a higher estimate than the accumulated cases reported in the entire Swedish population at a similar time. Our findings support the accumulation of knowledge suggesting that the risk that DMTs pose is smaller than previously presumed, however, our findings also suggest that PwMS, in general, are more vulnerable to infection. While further research on risk assessment for this population is still needed, tailored disease prevention and management should be considered for PwMS as a group at elevated risk given the likelihood of future pandemics.

## Supplemental Material

sj-docx-1-mso-10.1177_20552173241248293 - Supplemental material for Coronavirus disease 2019 infection among working-aged people with multiple sclerosis and the impact of disease-modifying therapies Supplemental material, sj-docx-1-mso-10.1177_20552173241248293 for Coronavirus disease 2019 infection among working-aged people with multiple sclerosis and the impact of disease-modifying therapies by Chantelle Murley, Emma Pettersson, Jan Hillert, Alejandra Machado and Emilie Friberg in Multiple Sclerosis Journal – Experimental, Translational and Clinical
