# Supplementary material for: Coronavirus disease 2019 infection among working-aged people with multiple sclerosis and the impact of disease-modifying therapies
Source: Mult Scler J Exp Transl Clin. 2024 Apr 27;10(2):20552173241248293. doi: 10.1177/20552173241248293 (PMC11055478; doi:10.1177/20552173241248293)
Supplement: sj-docx-1-mso-10.1177_20552173241248293 - Supplemental material for Coronavirus disease 2019 infection among working-aged people with multiple sclerosis and the impact of disease-modifying therapies [file sj-docx-1-mso-10.1177_20552173241248293.docx]

# Supplementary material

**Supplementary table 1: Changing the reference group for disease modifying therapy in the risk of COVID-19 (confirmed or suspected) analyses (Rituximab) among participants (n=3911)**

| **Characteristic** | Unadjusted | | Adjusted *^1^* | | Adjusted*^2^* | |
| --- | --- | --- | --- | --- | --- | --- |
|  | **OR** **(95% CI)***^3^* | **p-value** | **OR** **(95% CI)***^3^* | **p-value** | **OR** **(95% CI)***^3^* | **p-value** |
| **Latest DMT** |  |  |  |  |  |  |
| Rituximab | — |  | — |  | — |  |
| No treatment | 0.91 (0.68 - 1.20) | 0.50 | 0.92 (0.69 - 1.23) | 0.59 | 0.98 (0.73 - 1.31) | 0.90 |
| Alemtuzumab | 0.96 (0.47 - 1.82) | 0.90 | 0.94 (0.46 - 1.79) | 0.87 | 0.92 (0.45 - 1.75) | 0.80 |
| Cladribine | 0.71 (0.37 - 1.24) | 0.25 | 0.69 (0.37 - 1.22) | 0.23 | 0.68 (0.36 - 1.20) | 0.21 |
| Dimethyl fumarate | 1.07 (0.85 - 1.36) | 0.55 | 1.08 (0.85 - 1.36) | 0.53 | 1.05 (0.83 - 1.33) | 0.67 |
| Fingolimod | 0.99 (0.72 - 1.34) | 0.94 | 0.99 (0.72 - 1.34) | 0.94 | 0.97 (0.70 - 1.32) | 0.85 |
| Injectables | 0.90 (0.66 - 1.21) | 0.50 | 0.92 (0.68 - 1.25) | 0.61 | 0.91 (0.67 - 1.23) | 0.55 |
| HSCT | 0.61 (0.33 - 1.07) | 0.10 | 0.62 (0.33 - 1.09) | 0.12 | 0.64 (0.34 - 1.13) | 0.15 |
| Natalizumab | 0.98 (0.78 - 1.22) | 0.84 | 0.98 (0.78 - 1.23) | 0.87 | 0.97 (0.77 - 1.21) | 0.76 |
| Ocrelizumab | 1.01 (0.46 - 2.03) | 0.98 | 1.02 (0.46 - 2.05) | 0.97 | 0.99 (0.45 - 2.01) | 0.98 |
| Teriflunomide | 0.86 (0.53 - 1.35) | 0.52 | 0.87 (0.54 - 1.36) | 0.55 | 0.85 (0.52 - 1.33) | 0.49 |
| **Age (years)** |  |  |  |  |  |  |
| 20-29 | — |  | — |  | — |  |
| 30-39 | **0.77 (0.59 - 1.00)** | **0.049** | 0.79 (0.61 - 1.04) | 0.086 | 0.79 (0.61 - 1.04) | 0.091 |
| 40-49 | 0.81 (0.63 - 1.04) | 0.092 | 0.86 (0.67 - 1.11) | 0.25 | 0.88 (0.69 - 1.15) | 0.35 |
| 50-51 | 0.79 (0.57 - 1.10) | 0.17 | 0.84 (0.60 - 1.17) | 0.31 | 0.88 (0.63 - 1.24) | 0.48 |
| **Sex** |  |  |  |  |  |  |
| Women | — |  | — |  | — |  |
| Men | 1.05 (0.89 - 1.23) | 0.58 | 1.02 (0.86 - 1.19) | 0.84 | 1.02 (0.87 - 1.20) | 0.79 |
| **Country of birth** |  |  |  |  |  |  |
| Sweden | — |  | — |  | — |  |
| Other | 1.23 (0.99 - 1.53) | 0.057 | 1.16 (0.93 - 1.45) | 0.18 | 1.17 (0.94 - 1.46) | 0.16 |
| **Level of education** |  |  |  |  |  |  |
| Primary school | — |  | — |  | — |  |
| High school | **0.65 (0.47 - 0.91)** | **0.011** | **0.68 (0.49 - 0.96)** | **0.025** | **0.67 (0.48 - 0.95)** | **0.021** |
| University | **0.61 (0.45 - 0.85)** | **0.003** | **0.62 (0.45 - 0.87)** | **0.004** | **0.61 (0.44 - 0.85)** | **0.003** |
| **Type of living area** | |  |  |  |  |  |
| City | — |  | — |  | — |  |
| Town/suburb | **0.84 (0.72 - 0.98)** | **0.027** | **0.84 (0.71 - 0.99)** | **0.032** | **0.84 (0.72 - 0.99)** | **0.035** |
| Rural area | **0.75 (0.61 - 0.93)** | **0.007** | **0.74 (0.60 - 0.92)** | **0.006** | **0.75 (0.60 - 0.92)** | **0.008** |
| **Type of MS** | |  |  |  |  |  |
| Relapsing remitting MS | — |  |  |  | — |  |
| Primary progressive MS | 0.71 (0.38 - 1.24) | 0.25 |  |  | 0.72 (0.38 - 1.27) | 0.28 |
| Secondary progressive-MS | **0.70 (0.49 - 0.97)** | **0.040** |  |  | **0.68 (0.47 - 0.96)** | **0.032** |
| Missing | 0.72 (0.32 - 1.44) | 0.38 |  |  | 0.73 (0.33 - 1.47) | 0.40 |

*^1^* Mutually-adjusted for all other covariates except MS type.

*^2^* Mutually-adjusted for all other covariates including MS type

*^3^* Odds Ratio (OR) with 95% Confidence Intervals (CIs) from logistic regression models

***Abbreviations*:** CI: Confidence interval; DMT: Disease modifying therapy HSCT: Hematopoietic stem cell

transplantation; MS: Multiple sclerosis; OR: Odds ratio

**Supplementary table 2: Investigating the risk of COVID-19 among those reporting COVID-19 confirmed by a test result versus no COVID-19 (n=3630)**

| **Characteristic** | Unadjusted | | Adjusted *^1^* | | Adjusted*^2^* | |
| --- | --- | --- | --- | --- | --- | --- |
|  | **OR** **(95% CI)***^3^* | **p-value** | **OR** **(95% CI)***^3^* | **p-value** | **OR** **(95% CI)***^3^* | **p-value** |
| **Latest DMT** |  |  |  |  |  |  |
| Dimethyl fumarate | — |  | — |  | — |  |
| No treatment | 0.75 (0.51 - 1.10) | 0.15 | 0.75 (0.50 - 1.10) | 0.15 | 0.80 (0.54 - 1.19) | 0.28 |
| Alemtuzumab | 0.67 (0.26 - 1.48) | 0.35 | 0.67 (0.27 - 1.49) | 0.36 | 0.67 (0.27 - 1.48) | 0.36 |
| Cladribine | 0.72 (0.36 - 1.37) | 0.34 | 0.72 (0.35 - 1.36) | 0.33 | 0.72 (0.35 - 1.37) | 0.34 |
| Fingolimod | 0.79 (0.52 - 1.19) | 0.27 | 0.79 (0.52 - 1.19) | 0.27 | 0.79 (0.52 - 1.20) | 0.27 |
| Injectables | 0.86 (0.58 - 1.26) | 0.43 | 0.87 (0.58 - 1.28) | 0.48 | 0.87 (0.59 - 1.29) | 0.50 |
| HSCT | 0.52 (0.24 - 1.02) | 0.074 | 0.53 (0.25 - 1.04) | 0.081 | 0.56 (0.26 - 1.10) | 0.11 |
| Natalizumab | 0.89 (0.64 - 1.23) | 0.47 | 0.89 (0.65 - 1.24) | 0.50 | 0.90 (0.65 - 1.24) | 0.52 |
| Ocrelizumab | 0.96 (0.40 - 2.10) | 0.93 | 0.97 (0.40 - 2.13) | 0.94 | 0.97 (0.40 - 2.13) | 0.95 |
| Rituximab | 0.86 (0.66 - 1.12) | 0.25 | 0.86 (0.66 - 1.12) | 0.25 | 0.88 (0.68 - 1.14) | 0.32 |
| Teriflunomide | 0.53 (0.27 - 0.98) | 0.052 | 0.53 (0.27 - 0.97) | 0.051 | 0.53 (0.27 - 0.97) | 0.052 |
| **Age (years)** |  |  |  |  |  |  |
| 20-29 | — |  | — |  | — |  |
| 30-39 | 0.79 (0.59 - 1.07) | 0.12 | 0.81 (0.60 - 1.10) | 0.18 | 0.81 (0.60 - 1.11) | 0.18 |
| 40-49 | 0.85 (0.64 - 1.13) | 0.25 | 0.91 (0.68 - 1.22) | 0.51 | 0.93 (0.70 - 1.25) | 0.62 |
| 50-51 | 0.86 (0.60 - 1.25) | 0.44 | 0.92 (0.63 - 1.34) | 0.65 | 0.96 (0.66 - 1.40) | 0.83 |
| **Sex** |  |  |  |  |  |  |
| Woman | — |  | — |  | — |  |
| Man | 1.02 (0.85 - 1.22) | 0.83 | 1.00 (0.83 - 1.20) | 0.99 | 1.01 (0.84 - 1.21) | 0.94 |
| **Country of birth** |  |  |  |  |  |  |
| Sweden | — |  | — |  | — |  |
| Other | 1.17 (0.91 - 1.49) | 0.20 | 1.13 (0.87 - 1.44) | 0.36 | 1.13 (0.88 - 1.45) | 0.33 |
| **Level of education** |  |  |  |  |  |  |
| Primary school | — |  | — |  | — |  |
| High school | 0.76 (0.52 - 1.13) | 0.16 | 0.78 (0.54 - 1.17) | 0.22 | 0.78 (0.53 - 1.16) | 0.20 |
| University | 0.70 (0.49 - 1.03) | 0.066 | 0.71 (0.49 - 1.04) | 0.073 | 0.69 (0.48 - 1.03) | 0.061 |
| **Type of living area** | |  |  |  |  |  |
| City | — |  | — |  | — |  |
| Town/suburb | 0.88 (0.74 - 1.06) | 0.17 | 0.88 (0.73 - 1.05) | 0.16 | 0.88 (0.73 - 1.05) | 0.16 |
| Rural area | 0.80 (0.63 - 1.01) | 0.064 | **0.78 (0.62 - 0.99)** | **0.046** | **0.79 (0.62 - 1.00)** | **0.050** |
| **Type of MS** | |  |  |  |  |  |
| Relapsing remitting MS | — |  |  |  | — |  |
| Primary progressive MS | 0.70 (0.33 - 1.31) | 0.30 |  |  | 0.72 (0.34 - 1.36) | 0.34 |
| Secondary progressive-MS | 0.75 (0.50 - 1.08) | 0.13 |  |  | 0.73 (0.49 - 1.07) | 0.12 |
| Missing | 0.77 (0.31 - 1.65) | 0.54 |  |  | 0.78 (0.32 - 1.68) | 0.56 |

*^1^* Mutually-adjusted for all other covariates except MS type.

*^2^* Mutually-adjusted for all other covariates including MS type

*^3^* Odds Ratio (OR) with 95% Confidence Intervals (CIs) from logistic regression models

***Abbreviations*:** CI: Confidence interval; DMT: Disease modifying therapy HSCT: Hematopoietic stem cell

transplantation; MS: Multiple sclerosis; OR: Odds ratio
